# Operationalizing urgency in oncology: ethical challenges amidst the pandemic

**DOI:** 10.1007/s00432-024-05912-1

**Published:** 2024-09-04

**Authors:** Aaron Lawson McLean

**Affiliations:** Department of Neurosurgery, Jena University Hospital – Friedrich Schiller University Jena, Am Klinikum 1, 07747 Jena, Germany

**Keywords:** Ethical prioritization, Oncology care, Resource allocation, Patient engagement, Urgency criteria, Healthcare ethics

Dear Editor,

I read with great interest the recently published paper titled “What does ‘urgency’ mean when prioritizing cancer treatment? Results from a qualitative study with German oncologists and other experts during the COVID-19 pandemic” by Sommerlatte et al. (Sommerlatte et al. 2024). This insightful study examines the ethical complexities of prioritizing cancer care during periods of resource scarcity, an issue that has become critically relevant amidst the COVID-19 pandemic. The exploration of “urgency” as a criterion for prioritizing cancer treatment offers valuable contributions to the field of empirical bioethics and sheds light on the nuanced considerations necessary for effective and ethical decision-making in oncology.

The paper’s emphasis on distinguishing between different dimensions of “urgency” – namely, preventing imminent harm to life, preventing future harm, and alleviating suffering – is particularly noteworthy. This differentiation underscores the complexity of applying a seemingly straightforward criterion in the multifaceted context of cancer care. The study’s findings suggest that while “urgency” is a well-established criterion, its operationalization requires careful consideration of the specific circumstances and potential outcomes of each case. This approach aligns with the broader ethical principle of maximizing patient benefit while minimizing harm, yet it also highlights the inherent challenges in balancing these dimensions during a crisis.

One of the paper’s most compelling insights is the identified need to modulate “urgency” by considering the “success” and “likelihood” of success of an intervention. This triadic framework – urgency, success, and likelihood – offers a more comprehensive basis for prioritization decisions. It ensures that resources are allocated not only to those in immediate need but also to those for whom the intervention has a high probability of achieving significant benefit. This nuanced approach could mitigate the risks of oversimplification that often accompany urgent medical decision-making. This concept is reminiscent of the ethical theories proposed by Norman Daniels, who emphasizes the importance of fair equality of opportunity in healthcare (Skinner 2014).

However, the paper also reveals several limitations that merit further discussion. Firstly, the study’s focus on German oncologists and experts may limit the generalizability of its findings. Healthcare systems, cultural attitudes towards medical ethics, and resource availability can vary significantly across countries (Leijen and van Herk 2021). Thus, while the insights gained are valuable, their applicability to other contexts should be approached with caution. Comparative studies involving diverse healthcare settings would be beneficial in validating and extending these findings.

Furthermore, the study primarily engages with the perspectives of healthcare professionals and experts, potentially overlooking the patient’s voice. Patient-centered care is a cornerstone of modern oncology, and understanding patients’ perspectives on prioritization criteria, especially during crises, is crucial. Future research should incorporate patient and caregiver views to ensure that prioritization frameworks align with patient values and preferences. This holistic approach could enhance the ethical robustness and practical applicability of prioritization criteria.

The operationalization of “urgency” as described in the study also raises intriguing ethical questions about the implicit value judgments made during prioritization. For instance, prioritizing curative over palliative treatments inherently values potential life extension over quality-of-life improvements. This distinction, while clinically pragmatic, necessitates ongoing ethical scrutiny to avoid systematic biases against palliative care patients. An ethical framework that explicitly addresses these value judgments and incorporates a balanced consideration of both curative and palliative needs would be essential for fair and just prioritization.

Moreover, the study’s methodology, which combines qualitative interviews and group discussions, provides a rich, in-depth understanding of expert perspectives. Yet, this approach may also introduce certain biases, such as the dominance of more vocal participants in group discussions. Employing additional quantitative methods could help triangulate the findings and enhance their robustness. Mixed-methods research that integrates qualitative insights with quantitative data could offer a more balanced view and strengthen the evidence base for policy recommendations.

In conclusion, the study by Sommerlatte et al. significantly advances our understanding of ethical prioritization in oncology during resource-constrained periods. The proposed framework of urgency, success, and likelihood offers a nuanced and ethically sound approach to decision-making. However, the generalizability of the findings, the need for patient-centered perspectives, and the ethical implications of value judgments in prioritization warrant further exploration. Comprehensive and reflective studies such as this are indispensable in guiding medical practice and policy amidst ongoing and future challenges in global healthcare.

Sincerely,

Aaron Lawson McLean

## Data Availability

All data analyzed are contained within the manuscript. No additional data were generated during the course of this study. All relevant results and evidence supporting the conclusions are available to readers, and no supplementary data is required.
